# Current Diagnostic Methods for Assessing Transfer of Passive Immunity in Calves and Possible Improvements: A Literature Review

**DOI:** 10.3390/ani11102963

**Published:** 2021-10-14

**Authors:** Rayanne Soalheiro de Souza, Lucas Braga Costa dos Santos, Isabela Oliveira Melo, Daiane Maria Cerqueira, Juliana Vieira Dumas, Fabiola de Oliveira Paes Leme, Tiago Facury Moreira, Rodrigo Melo Meneses, Antônio Ultimo de Carvalho, Elias Jorge Facury-Filho

**Affiliations:** Department of Veterinary Clinic and Surgery, Veterinary School, Universidade Federal de Minas Gerais, Belo Horizonte 31270-901, MG, Brazil; ra.soalheiro@vetufmg.edu.br (R.S.d.S.); lucasbragacosta2@gmail.com (L.B.C.d.S.); isabelamelovet@gmail.com (I.O.M.); daianecerqueira@yahoo.com.br (D.M.C.); julianadumas5@gmail.com (J.V.D.); fabiolapaesleme@vetufmg.edu.br (F.d.O.P.L.); tiagofacuryvet@gmail.com (T.F.M.); menesesrm@gmail.com (R.M.M.); ultimovet@gmail.com (A.U.d.C.)

**Keywords:** immunoglobulins, analysis methods, electrophoresis, monitoring, gold standard method

## Abstract

**Simple Summary:**

The transfer of passive immunity (TPI) from cows to calves needs to be routinely assessed on farms and in field research. The gold standard method for assessing TPI is radial immunodiffusion (RID) because it directly assesses the immunoglobulin G (IgG) concentration in calf serum samples. In addition to RID, there are several other methods available for the assessment of TPI which determine the concentration of IgG or other serum components. It is known that several components present in the colostrum are absorbed by the calves and aid passive immunity. We conducted a literature review of the methods scientifically reported by experts in the field.

**Abstract:**

Several direct or indirect methods can be used to assess immunoglobulin G (IgG) concentrations in calves, which evaluates the transfer of passive immunity (TPI). Radial immunodiffusion (RID) is the gold standard method to measure serum IgG in bovines. Previous studies have shown that colostrum provides several molecules in addition to immunoglobulins, which play an important role in the passive immunity of the calf. However, no studies have yet determined the level of interference of these components in the immunity, health and survival of calves. In this sense, the objective of this study is to review the methods of evaluation available for the laboratory and field diagnosis of TPI in calves and discuss the main aspects of each technique. Several methods available for TPI evaluation in calves may provide insights into the various components of colostrum involved in passive immunity.

## 1. Introduction

The transfer of passive immunity (TPI) is defined as the absorption of the maternal immunoglobulin present in colostrum through the small intestine of the calf during the first 24 h after birth [1]. TPI is frequently evaluated using direct or indirect doses of immunoglobulin G (IgG) and radial immunodiffusion (RID) is considered the gold standard method for this purpose [2]. Several scientific groups are researching other strategies to directly or indirectly evaluate bovine IgG.

Some of the tests were developed with the goal of being field-performed (point-of-care tests [POC]) directly on farms. IgG could be directly evaluated using turbidimetric immunoassays [3,4], an enzyme-linked immunosorbent assay (ELISA) [5], electrophoresis [6,7,8], capillary electrophoresis (CE) [9,10], the transmitted attenuated total reflectance infrared (ATR) spectroscopic method [11,12], a split trehalase IgG assay (STIGA) [13], and other innovative methods such as the liquid atmospheric pressure matrix-assisted laser desorption/ionization by mass spectrometry (AP-MALDI-MS). For indirect immunoglobulin measuring, the biochemical analysis of the total proteins and fractions [14], total protein measurements using refractometry [6,15,16,17,18,19,20], BRIX using refractometry [15,18,19,20,21,22], the zinc sulfate turbidity test [7,14,23], and the sodium sulfite turbidity test [7,16] are also available. Another method related to colostrum management is the biochemical analysis of the newborn calf serum, gamma glutamyltransferase (GGT), [1,14,24] since it allows for the evaluation of colostrum intake.

Hence, there are several methods for assessing IgG concentration, but several studies on TPI use different techniques, making a comparison between studies difficult. Other components of the colostrum are also absorbed in calf intestines and may also play an important role in passive immunity [1,8,25]. The aim of this review is to analyze the evaluation methods available for the laboratory and field diagnosis of TPI in calves, discuss the positive and negative aspects of each technique and suggest possible improvements in the evaluation of the TPI in the future.

## 2. Why Assess TPI?

An inadequate colostrum supply leads to the failure of the transfer of passive immunity (FTPI). FTPI is defined as a <1.0 g/dL serum IgG concentration in 1–7 d old calves [16]. This cutoff point is questioned and higher cutoffs, such as 1.5 [26,27] or 2.0–2.5 [28] g/dL, which are more consistent with the challenges posed by the current rearing systems and related to better calf performance, are also suggested. Recently, Lombard et al. [29] reviewed the cutoff points for TPI and suggested the new cutoff points based on the increasing IgG and serum total protein (STP) concentrations, which were related to a reduced morbidity risk and improved calf performance. The authors proposed four categories for individual calves and herd-based evaluations. The category of Excellent TPI needed to achieve ≥2.5 g/dL of IgG, 6.2 g/dL of STP or 9.4% BRIX in at least 40% of the herd. The category of Good TPI considered the calves that had between 1.8 and 2.49 g/dL of IgG, 5.8 and 6.1 g/dL of STP or 8.9 and 9.3% BRIX in approximately 30% of the herd. The category of Fair TPI considered the calves that presented between 1.0 and 1.79 g/dL of IgG, 5.1 and 5.7 g/dL of STP or 8.1 and 8.8% BRIX in approximately 20% of the herd. Finally, the category of Poor TPI considered the calves that had less than 1.0 g/dL of IgG, less than 5.1 g/dL of STP or less than 8.1% BRIX in no more than 10% of the herd in that category.

FTPI was related to a high mortality and morbidity risk, damaging the production system [30,31,32,33]. Additionally, FTPI could reduce milk yield during the first lactation, thus these animals were likely to be slaughtered due to low milk yield [34]. Raboisson et al. [35] estimated the average total cost per dairy calf with FTPI as EUR 60 (EUR 10–109), ranging from EUR 52 (best case scenario) to EUR 285 (worst case scenario), with an increase of approximately 50% for beef calves. Thus, FTPI also has a major economic impact on calf rearing systems.

FTPI prevalence in the United States is estimated to have decreased from 40% in 1991–1992 to 19.7% in 2007 [36], and to 12.1% in 2014 [37], possibly due to the improved management of calf rearing systems to monitor TPI and correction of failures in this regard. In Canada, Italy, Australia, and New Zealand, the prevalence of FTPI is currently 37.1–43.3% [15,38], 41% [39], 36.2–41.9% [40,41], and 25% [42], respectively. Due to the production, health, and economic impacts of FTPI and its high prevalence in several countries, this problem should be seriously addressed worldwide [39].

TPI monitoring identifies management failures in order to correct them, thus improving the health of calves [43]. According to Beam et al. [36] FTPI is more prevalent in farms that do not routinely monitor TPI, and are 13.8 times more likely to have FTPI in calves than farms that perform this monitoring. Obviously, simply monitoring TPI does not reduce FTPI in calves, but suggests that colostrum management and improvements that favor adequate TPI in calves are relevant to that herd. Therefore, to ensure that calves are adequately immunized and colostrum management is effective, it is necessary to directly or indirectly dose IgG in calf serum.

## 3. Methods of Evaluating TPI

Table 1 summarizes the TPI assessment methods by classifying them according to the IgG evaluation method, operation principle, main characteristics, statistics compared to the gold standard, and the possibility of using these methods in the field. Next, we analyzed each method individually.

### 3.1. Direct Methods

#### 3.1.1. RID

RID determines the concentration of a particular antigen or antibody in an unknown sample. Thus, RID assays are still used for certain applications in human and veterinary medicine, but they are usually low-volume assays in specialized centers [47]. In veterinary studies, RID was used for a long time as the gold standard to measure IgG in bovine colostrum and serum to evaluate TPI in calves [6,48,49,50].

RID is a classic method performed according to the technique described by Mancini, Carbonara, and Heremans [51]. Briefly, 3 µL serum sample and control are deposited in separate wells of a plate agarose gel sold with a bovine IgG-specific antiserum. The serum samples and control antigens diffuse into the gel and a precipitating ring is formed after 18–24 h with a diameter proportional to the IgG concentration. Hence, the IgG concentration for a particular diameter can be obtained after drawing a standard curve [52]. RID is pointed out in the literature as expensive as it requires specialized labor for its realization and uses reagents and antibodies with a limited durability [20]. Technically, the test has limitations in the samples which form very large or very small ring diameters. However, in these cases, it is possible to dilute or concentrate the serum samples and repeat the examination [5]. RID can present high-variance inter assays [6]. Accordingly, it is common to perform the sample analysis at least twice and repeat the analysis if the coefficient of variation between the samples is higher than 10% [13,51]. The possible implications of test repetition are delayed in the results and make the analysis more expensive.

As RID is a direct semiquantitative test of bovine IgG, it is considered the gold standard, that is, it is the reference test that serves as a comparison for other tests to confirm FTPI [1,2]. However, it requires a minimum of 18–24 h to obtain the results and its use is not recommended as a routine in farm monitoring for a TPI assessment [10,19,20]. Thus, the use of RID is recommended only to investigate and study FTPI-related mortality outbreaks [53].

A study comparing two commercial RID tests was performed by evaluating 30 blood samples collected from newborn holstein calves at birth and 24 h after colostrum ingestion, with one commercial bovine serum used as a reference and two different purified bovine IgG concentrations [52]. The results of the two kits were highly discrepant and showed a poor agreement which could be due to inaccuracies in the internal standards of the kits. Some studies also demonstrated that RID showed higher values of IgG than those obtained from using other direct methods, such as ELISA and electrophoresis [5,6,9,44].

Despite this, RID was used for many years as the gold standard in research on TPI in calves.

#### 3.1.2. Turbidimetric Immunoassays

The immunoassay used to detect serum or plasma IgG concentrations in calves took approximately 10 min to determine the IgG concentration by reading the absorbance at 340 nm using a spectrophotometer [3]. Two versions of the immunoassay-based rapid tests were commercially available, one of which semi-quantitatively assessed the TPI and another which quantitatively assessed the IgG concentration.

A semiquantitative test (ZAPvet Bovine IgG test; NOWDiagnostics; Toronto, ON, Canada) was developed using calibrators with concentrations determined by a turbidimetry. It used two visual lines, a test line and a reference line, to estimate the bovine IgG concentration. To perform the test, 35–40 μL of whole blood, plasma, or serum was placed in the sample application zone on a flat surface, incubated at room temperature for 15 to 20 min, and then read by comparing the test line intensity with the reference line intensity [4]. Renaud et al. [54] compared the results of 149 Holstein calves using this semiquantitative rapid test and RID and observed 77% sensitivity and 44% specificity using RID as the gold standard. The authors attributed this result to the slight changes in the test line intensity from the reference line intensity and the difficulty of depositing the correct amount of sample in the application zone.

Alley, Haines, and Smith [3] used a quantitative, commercially available immunoassay (bovine serum/plasma IgG; Midland BioProducts Corp.; Boone, IA, USA) and a portable analyzer (MBC QTII; Midland BioProducts Corp.; Boone, IA, USA) and quantified the results of 100 blood samples collected from 1–5 d old calves. The authors observed that the coefficient of variation of the turbidimetric immunoassay technique was similar to that of RID (47.2 and 47.1%, respectively) with a correlation coefficient between the two techniques of 0.988. However, semiquantitative and quantitative turbidimetric immunoassays tests were quick and simple, which favored their use on farms [3,54].

#### 3.1.3. ELISA

ELISA is a semiquantitative immunoglobulin concentration test. Like RID, ELISA is a laboratory test and has the same practical limitations, although it is cheaper than RID due to its greater availability [5].

Lee et al. [46] evaluated 115 0–10 d old calves and observed a 94% agreement with a 0.98 area below the curve, 0.98 sensitivity, and 0.91 specificity between the results obtained from RID and ELISA. However, Gelsinger et al. [5] evaluated 104 blood samples from 48 h old beef calves and found that ELISA showed consistently lower values than RID. They suggested that this large variation could be inherent to ELISA, as its methodology required several dilutions, whereas RID required a minimal sample dilution. In addition, a large proportion of the samples required a repeat analysis when tested using ELISA than when using RID. Similarly, Dunn et al. [44] analyzed 10 48 h old calf and beef calf serum samples and found that, although there was a high correlation between RID and ELISA (R^2^ = 0.97; *p* < 0.001), on average, the IgG values determined using RID were 1.8 higher than those determined by ELISA.

Although ELISA is widely used for the determination of bovine IgG concentration in laboratories, it is not highly recommended for use due to the delay in obtaining the results.

#### 3.1.4. Transmitted and Attenuated Total Reflectance Infrared (ATR) Spectroscopic Method

There are two methods for analyzing infrared (IR) spectra: transmission and attenuated total reflectance (ATR), which suit TPI evaluation in calves for the possibility of field use [11]. The measurement is simple, fast, and requires little or no sample preparation [11].

To evaluate ATR–IR as a technique for determining the IgG concentration in calves for TPI evaluation, the results of 208 calf samples were compared using ATR–IR and RID. The sensitivity, specificity, and correlation coefficient of the ATR–IR were 0.92, 1.0, and 0.93, respectively, suggesting that it could be used in place of the gold standard technique, with the advantage of being portable and fast, enabling the analysis in the field [11]. In another study, the accuracy of ATR–IR was 0.93 [12]. Subsequently, other studies were performed using the IR spectroscopy technique for TPI assessment, one of which found that the correlation was 0.92 in 217 3–10 d old calves [15]. Another study compared the transmission IR spectroscopy with a BRIX refractometer (8.2% BRIX cutoff) in 691 samples from 1–14 d old calves, with a 0.79 sensitivity and 0.97 specificity [15]. However, the high cost of the equipment was a strong limiting factor for the diffusion of this technique.

#### 3.1.5. STIGA

STIGA is also developed as a rapid test for measuring IgG on farms [13]. It utilizes the trehalase glycolytic enzyme (TreA, a trehalose-to-glucose converter). TreA is divided into two non-functional fragments, TreAN and TreAC, both fused to the streptococcal protein G. The Protein G specifically binds to the IgG constant region (Fc) and acts as an IgG sensor regardless of the antigen binding specificity. When the two fusion proteins are incubated with samples containing IgG (either colostrum or serum), both bind to IgG Fc, leading to TreA dimerization and reactivation. Reactivated TreA converts trehalose to glucose, which can be quantified using colorimetric assays or glucometers, and this case can be read in the field [13].

Drikic et al. [13] reported that the correlation coefficient between the IgG determined using RID and that using STIGA by the colorimetric technique was 0.9 and 0.85 for dairy and beef calf samples, respectively. STIGA also correctly identified 95 and 75% of FTPI cases in dairy and beef calf serum samples, respectively, and 90 and 94.5% of adequate TPI in dairy and beef calf serum samples, respectively. Thus, this technique has a high potential to directly analyze IgG in calves for field use but, to date, it is not commercially available.

#### 3.1.6. Electrophoresis

Electrophoresis is the most commonly used analytical technique for serum or plasma protein fractionation in the laboratory [55]. It is very common and can be performed in many institutions, but it is unlikely to be used in the field because of many challenges. Electrophoresis is based on the movement of charged protein molecules in an electric field [6,55]. After the gel run, it is possible to quantify the proteins in an electrophoretogram and thus determine the concentration of each protein fraction in the serum or plasma sample analyzed [56].

Albumin is a mostly negatively charged and small molecule, making it one of the most mobile proteins in the electrophoresis gel. Immunoglobulins, particularly gamma globulins, are the least negatively charged and migrate slowly. The method involving these molecules has a higher accuracy for IgG evaluation (89%) [6] than RID. Previous studies, using serum samples from foals [57] and donkey foals [58], compared RID with electrophoresis. A total of 360 serum samples from 60 foals within 6 to 24 h of life were analyzed [57] and showed a good agreement between IgG concentrations using RID and gamma globulin concentrations using electrophoresis (Bland–Altman systemic bias: −1.9 g/L; 95% CI −2.4–−1.5). Turini et al. [58] analyzed serum samples at 0, 6, 12, and 24 h of life for nine donkey foals, and observed a strong significant correlation between serum IgG using RID and STP by electrophoresis (r = 0.87; *p* < 0.0001) and a good significant correlation between serum IgG by RID and serum gamma globulin by electrophoresis (r = 0.56; *p*-value between 0.01 and 0.001). 

Electrophoresis can be an interesting method for the laboratory analysis of TPI because it is faster and more available in laboratories than RID. In addition, it is possible to separately evaluate the increase in each fraction of the absorbed protein. Given the increase in the attention to other colostrum components over the past few years, this characteristic makes electrophoresis an interesting tool. However, specific cutoff points must be generated for the technique so that accurate results can be generated for a TPI evaluation in neonatal calves [9].

#### 3.1.7. Capillary Electrophoresis (CE)

CE is a separation method in which charged species are separated based on charge and size by their different rates of migration in an electric field. CE is routinely used in human and animal clinical laboratories to screen for abnormal serum protein profiles [59,60,61]. In addition, CE presents a minimum operation cost, simple instrumentation, does not require the intensive training of technicians (i.e., fully automated), presents a high efficiency of separation, short run time (approximately 20 min compared with almost 24 h with the RID assay), and compatibility with small sample volumes [10,61]. Furthermore, CE detects the monoclonal components (immunoglobulins) present in the gammaglobulin fraction. In this sense, CE is used to evaluate colostrum and TPI in bovines and other ruminants [9,10,62].

Morittu et al. [10] reported a polynomial trend between the results of the RID and CE analyses (RID = 0.02CE² − 0.04CE + 4.13; coefficient of determination, R² = 0.96; *p* < 0.05), suggesting a strong relationship between these methods when evaluating TPI in lambs. In addition, this study showed high levels of agreement between the RID and CE methods (bias: −0.04 g/L; *p* > 0.05; agreement limits: −6.38 g/L [low] and +6.30 g/L [high]). Sutter et al. [9] compared RID with capillary electrophoresis using 216 serum blood samples from 1–7 d old calves and observed a 0.97 correlation between these tests, but, on average, IgG concentrations measured using RID were 0.61 g/dL higher than those measured using CE.

The studies mentioned above show that CE is a suitable method for a reliable estimation of IgG and, consequently, the evaluation of TPI in calves. Furthermore, CE may have the shortest time between the test request and result release (turnaround time) compared to the other TPI evaluation methods.

#### 3.1.8. Proteomics

Proteomic studies separate and individually quantify the proteins present in tissue or fluid samples [55]. Two-dimensional gel electrophoresis, mass spectrometry, and non-gel-based proteomic analysis, such as surface-enhanced laser desorption ionization mass spectrometry (SELDI–MS), could be used for this purpose [55]. The proteome was a set of proteins expressed by a genome and could vary according to the moment and physiological conditions that the sample was submitted to [63]. Zhang et al. [64] identified 212 different types of proteins in the colostrum and milk proteome of four cows and quantified 208 of the identified proteins. Mann et al. [65] identified 663 different kinds of proteins, while using proteomics techniques to evaluate the serum proteins with a low abundance in newborn calves. However, although Mann et al. [65] measured IgG using RID, the authors did not quantify IgG using the proteome. To the best of our knowledge, data from comparing IgG results using proteomics and RID techniques are lacking.

The techniques for conducting proteomics studies are still expensive and restricted to some laboratories, although their use is increasing. We believe that this analysis can be fairly illuminating in the understanding of TPI in calves due to the results already observed in colostrum and milk evaluation.

#### 3.1.9. Liquid Atmospheric-Pressure Matrix-Assisted Laser Desorption/Ionization Using Mass Spectrometry (AP-MALDI-MS)

Matrix-assisted laser desorption/ionization (MALDI) is an ionization technique for the analysis of biomolecules using mass spectrometry (MS) [66]. Liquid MALDI allows for the generation of predominantly multiply charged ions under atmospheric pressure (AP) MALDI ion sources for MS analysis [66].

Piras et al. [67] used liquid AP-MALDI-MS to evaluate the profiling of lipids and proteins from the milk samples of goats (*n* = 43) and sheep (*n* = 44) for the speciation with an 100% accuracy in 1 minute of data acquisition per sample. This study also used liquid AP-MALDI-MS to evaluate the colostrum samples from sheep (*n* = 16). Samples were collected at 0 h (within six h of parturition) and 48 h after the first collection. This method could distinguish between the colostrum collected within 6 h after parturition and that which was collected 48 h later from the same animals with an accuracy of 84.4%. In a study using liquid AP-MALDI-MS for the diagnosis of mastitis in 135 milk samples from cows, Hale et al. [68] showed that this method provided a greater diagnostic of mastitis information (total classification accuracy of 95.56%). The authors considered that this method had a potential diagnostic applicability. To the best of our knowledge, no studies reported using AP-MALDI-MS to assess TPI in calves, which could reveal an opportunity for scientific advances in this field.

This method is easy to operate, rapid and robust, in addition to enabling a low-cost analyses using crude bioliquids [67,68]. Thus, this method is an affordable, innovative, and promising method for TPI evaluation.

### 3.2. Indirect Methods

#### 3.2.1. Biochemical Analysis of Total Proteins and Fractions

Newborn calves have an interesting serum protein profile. Some protein fractions are stable in serum samples despite the colostrum intake. For example, the concentration of albumin, the most prevalent STP, constituting 35–50% STP, tends to be constant in the blood of newborn calves [55]. In newborn calves, albumin concentrations are almost 2.5–3.0 g/dL [69,70]. Albumin tends to be stable in animals (unless severely dehydrated) as it participates in osmotic pressure maintenance and metabolite transport, which are fundamental to the survival of the individual [15,55]. However, other STP fractions vary according to the colostrum intake in newborn calves. The main example of these fractions is immunoglobulins. Calf are hypogammaglobulinemic at birth [2]. This means that almost all of the immunoglobulins found in the serum of a newborn calf until 21–28 d after birth are maternal immunoglobulins absorbed from the colostrum [71]. The STP also consists of other components, which are discussed later in Section 4.1. In general, STP can be divided into two main fractions: albumin and globulins. Since albumin has a certain stability and newborn calves do not experiences many deficiencies which could cause variations in other proteins, there is a strong correlation between STP and the absorbed colostrum immunoglobulins in these animals during the first days of their lives. This correlation allows the STP analysis to indirectly evaluate IgG and, consequently, TPI [16,17,72]. For instance, Denholm et al. [22] evaluated 101 samples from 1–7 d old calves and demonstrated that a 5.6 g/dL STP concentration using the biuret method was equivalent to a 1.0 g/dL IgG concentration using the RID method.

An important aspect of the total protein measuring to assess TPI was the calf age. Nocek et al. [73] evaluated 159 calves on different diets from colostrum up to 45 days of age and demonstrated a significant positive correlation (r = 0.84, *p* < 0.001) within 12–24 h after birth, and an equal result on day 4. On day 11, the correlation was maintained, but it reduced (r = 0.69), suggesting that age influenced IgG determination. This phenomenon was most likely related to protein catabolism.

This information was very relevant, as it influenced the required period of the TPI assessment on the farm. Wilm et al. [72] showed that up to 9 d old calves could be safely tested for TPI using IgG or STP concentrations because, compared to the IgG or total protein value at 24 h (reference from the same individual), STP concentrations were correlated in 2–3 d old calves (r ≥ 0.98); correlated, but variable in 4–9 d old calves (r ≥ 0.88); and slightly correlated in 10 d old calves (r = 0.76). However, these authors warned that all evaluated individuals remained healthy throughout the study and none had FTPI, thus not reflecting the reality of most farms, and further studies were needed to confirm whether these results were also valid for calves who became ill in the first weeks of age and/or have FTPI. Thus, to obtain more reliable information regarding TPI, it was ideal to collect serum samples for 24 to 48 h, if feasible.

In a study by Denholm et al. [22], the STP evaluation using the biuret method was more accurate than an evaluation by the refractometry method when compared to RID (accuracy = 83.1% vs. 69.3%, respectively). Since an increasing number of farms were monitoring TPI in calves using protein refractometers [37], laboratory STP evaluation using biochemical analysis could be an important method for the periodic assessment of the accuracy of the equipment being used on the farm.

#### 3.2.2. Protein Refractometry

The optical or digital protein refractometer measures the amount of light refracted due to the serum sample constituents [74]. For this, a light beam must pass through the serum sample deposited in the prism. In the serum, the proteins refract light and the amount of light refracted increases with the increase in protein content [17]. Refractometer use is based on the fact that low STP concentrations are associated with FTPI [7,29,33].

Using a manual refractometer has several advantages in monitoring colostrum management, as it is a cheap, simple, and fast tool that can be used on the farm [53]. However, its results should be interpreted carefully as factors such as equipment quality, maintenance and calibration, sample temperature, and calf age may influence the results [2,17].

Buczinski et al. [75] concluded that to minimize the number of false negative cases (i.e., the number of calves with FTPI not detected by the test), a cutoff point of 5.5 g/dL instead of 5.2 g/dL for the total protein refractometry seemed to be an appropriate threshold for ruling out FTPI when reviewing studies involving the total protein refractometers. Elsohaby et al. [76] determined the serum and plasma cutoffs to be 5.1 and 5.8 g/dL, respectively, using 217 3–10 d old calves. Thus, the analysis could be performed with both types of samples as long as the cutoff point for the TPI analysis was appropriate. The cutoff point could also be adjusted on the farm to improve the colostrum management parameters and avoid failures associated with equipment errors. Recently, a group of calf experts from the USA and Canada revised the cutoff points for TPI categorizing individuals and herds, as already commented [29]. Thus, the cutoff points currently considered for the Excellent, Good, Fair and Poor TPI categories using the protein refractometer were greater than 6.2 g/dL of STP (at least 40% of the herd), between 5.8 and 6.1 g/dL of STP (approximately 30% of the herd), between 5.1 and 5.7 g/dL of STP (approximately 20% of the herd), and less than 5.1 g/dL of STP (no more than 10% of the herd), respectively.

Lee et al. [46] observed a 73% sensitivity and 78% specificity in 115 0–10 d old calf serum samples, using cutoffs of 5.8 and 1.0 g/dL for STP in the refractometer and IgG in RID, respectively. The correlation coefficients between the STP refractometry and RID were 0.49–0.93 [17,19,53]. The cause of variation between the studies may be related to differences in the refractometry technique and the age of the animals. The optical refractometer is not accurate enough to determine small differences, and most refractometers are accurate to 0.2 g/dL. This means that they are not very effective in distinguishing between 5.0 and 5.2 g/dL, for example, which can cause problems in detecting TPI, particularly if they do not undergo periodic evaluations. Despite this, protein refractometers are interesting tools for TPI evaluation on farms because they are portable, cheap, simple, and practical to use.

#### 3.2.3. BRIX Refractometer

Similar to total protein refractometers, BRIX refractometers (digital or optical) determine the serum BRIX score by passing a light beam through the sample to the prism, measuring the refractive index, and reading it on a scale (% BRIX). The BRIX refractometer measures sucrose concentration in liquids, such as fruit juice, molasses, and wine. However, when used in liquids without sucrose, the BRIX percentage (% BRIX) approximates the total solid percentage [77]. It is an extremely useful tool in practice because, in addition to being used in TPI evaluation, it can also be used to evaluate colostrum quality, allowing for the two-point monitoring of colostrum management with a single device [19,78].

Morrill et al. [18] evaluated the serum of 200 calves on the first day of life using both the BRIX digital refractometer and IgG measurement by RID, suggesting 7.8% BRIX as a cutoff point for identifying FTPI. Elsohaby et al. [15] also compared these methods and suggested 8.3% as a cutoff point after evaluating 691 1–14 d old calves. Thornhill et al. [79] analyzed samples from 2 d old calves and determined the cutoff point to be 10% for both optical and digital BRIX refractometers and RID. Possible reasons for these differences may be related to using refractometers from different manufacturers, as well as the age, breed, and health status of the calves involved in the studies [76]. For the new categorization for TPI assessment, Lombard et al. [29] suggested that 8.1% BRIX was equivalent to FTPI. This wide variation between cutoffs created an uncertainty in using this equipment, but it was still the most widely used tool in the field to monitor TPI because of its speed, ease of use, and low cost [53].

To assess colostrum quality, a cutoff point of 22% BRIX was determined for both optical and digital refractometers, as a BRIX optical refractometer had 88.7 and 91.1% sensitivity and 75 and 91.7% specificity for heifers and cows, respectively, while a BRIX digital refractometer had 90.1 and 92.2% sensitivity and 37.5 and 83.3% specificity for heifers and cows, respectively [78].

#### 3.2.4. Zinc Sulfate Turbidity Test

To perform the zinc sulfate turbidity test, a solution containing 208 mg ZnSO_4_ ·7H_2_O/L distilled water was prepared and boiled to remove CO_2_. The sample (100 µL) was homogenized with 6 mL solution and incubated for 1 h. The absorbance at 660 nm was measured using a spectrophotometer and the turbidity was converted to mg IgG/L after producing a standard curve derived from samples with known bovine IgG concentrations or performed visually [6]. For the visual assessment, 200, 250, 300, 350, and 400 mg/L ZnSO₄ solutions were prepared. The test results were recorded as 0, 1, 2, 3, 4, or 5, where (0) equals no turbidity at any concentration; (1) equals turbidity only at 400 mg/L; (2) equals turbidity at 350 and 400 mg/L; (3) equals turbidity at concentrations of 300, 350 and 400 mg/L; (4) equals turbidity at 250, 300, 350 and 400 mg/L; and (5) equals turbidity at all five concentrations [46].

Hogan et al. [14] observed a 98.63% sensitivity and 30.19% specificity of the ZnSO₄ turbidity test, compared with that of RID, in serum samples from 126 48 h old calves and 70% calves were correctly classified as FTPI. Lee et al. [46], using the visual evaluation of this technique, observed a 68% sensitivity and 97% specificity of the ZnSO₄ turbidity test, compared with that of RID (cutoff point: 1.0 g/dL IgG), and reported that the ZnSO₄ turbidity test was a useful screening device with a high specificity, but recommended a confirmatory IgG concentration diagnosis with a direct measurement, such as RID. Pfeiffer et al. [6] argued that the ZnSO₄ turbidity testing was easy to perform and inexpensive, but was affected by factors such as sample hemolysis, reaction time, ambient temperature, and CO_2_ acting on the ZnSO₄ solution.

#### 3.2.5. Sodium Sulfite Turbidity Test

The sodium sulfite turbidity test uses different solutions that selectively precipitate high-molecular-weight proteins, including immunoglobulins, which help to evaluate turbidity [7].

For the sodium sulfite turbidity test, 14, 16, and 18% sodium sulfite solutions were prepared using distilled water. For each serum sample, three borosilicate test tubes (13 × 100 mm) containing 1.9 mL of each solution were prepared. Then, 0.1 mL serum was added to each of the three tubes, mixed, and incubated for 15 min at 23 °C. The turbidity of the tubes was observed and they were classified in a cross scale: 0, 1+, 2+, 3+, where 0 was equivalent to no turbidity in all three tubes, 1+ was equivalent to turbidity observed in 18% sodium sulfite solution, 2+ was equivalent to turbidity observed only in the sodium sulfite solutions of 16% and 18%, and 3+ was equivalent to turbidity observed in all three tubes [16]. Increasing the reagent or saline concentrations induced turbidity in the samples with reduced concentrations of high-molecular-weight proteins [7].

According to Howard [80], the result was interpretated as: 3+: IgG concentration >1.5 g/dL, indicating adequate immunization; 2+: IgG concentration 0.5–1.5 g/dL, indicating partial FTPI; 1+: IgG concentration <0.5 g/dL, indicating FTPI; and 0: FTPI.

Tyler et al. [16] observed that the cutoff point of the sodium sulfite turbidity test using the two lowest dilutions, 14 and 16%, corresponded to the excessively high IgG concentrations in 242 1–8 d old calves (in this study, 1+, 2+, and 3+ corresponded to 1.250, 2.116, and 2.948 g/dL serum IgG, respectively). According to this study, using 14 or 16% test solution tended to misclassify large numbers of calves with adequate serum immunoglobulin concentrations as FTPI. Although the results could be affected by calf age, some researchers advised against using this method for evaluating TPI [2,53]. However, Lee et al. [46] compared the three-tube sodium sulfite test (14, 16, and 18%) results to the RID results in 115 0–10 d calves, and considered 1.0 g/dL as the IgG concentration cutoff point, and an observed area below the curve, the sensitivity, and specificity to be 0.88, 76, and 99%, respectively, thus suggesting that the three-tube sodium sulfite test was superior to the other indirect methods compared in the study (total protein by refractometry, glutaraldehyde coagulation, and zinc sulfate turbidity). In addition, it was one of the fastest and cheapest techniques in the study, with a maximum time of 1 h and USD 0.57/unit cost. Thus, sodium sulfite turbidity testing could be an effective, rapid, simple, inexpensive, and indirect method for the assessment of TPI in the field, particularly in small farms with few calf births, where the purchase of a refractometer may not be suitable.

### 3.3. Another Way to Evaluate Colostrum Intake–γ Glutamyltransferase (GGT)

The mammary gland ducts in calves produce GGT during colostrogenesis (up to 30,540 U/L in colostrum), which decreases until 7 d after colostrum milking (3120 U/L in milk) [81]. When the calf ingests colostrum, GGT is absorbed and its activity can be detected in the serum. The serum GGT activity in the colostrum-fed calves is 60–160 times greater than that in healthy adult cattle [24].

Hogan et al. [14] compared the GGT values in 126 samples of 48 h calf serum (100 IU/L GGT cutoff) with those observed in RID and observed a 97.26% sensitivity and 98.11% specificity (FTPI cutoff point: 1.0 g/dL IgG in RID); 98% calves were correctly classified as having an adequate TPI. Güngör, Bastan, and Erbil [24] observed a significant (*p* < 0.05) but moderate positive correlation between the serum IgG concentrations obtained by RID and the serum activity levels of GGT in serum samples from 40 1 d old calves (r = 0.566), indicating large variations between the studies. These results were possibly associated with the quality of the colostrum supplied to the calves, because colostrum GGT concentration had no biological relation with the colostrum IgG concentration. Thus, a calf with a high serum GGT concentration did not indicate the ingestion of good-quality colostrum, only that the colostrum was ingested. Thus, the most appropriate interpretation for the test was that the serum GGT indicated colostrum ingestion but did not allow for the accurate serum IgG concentration assessment, and thus did not provide direct information on TPI [2].

Serum calf GGT dosage can be used strategically to identify colostrum delivery failures, especially when it is unclear whether the calf is actually receiving colostrum or suckling from their mothers on farms where deliveries do not occur (for example, beef cattle farms). However, calves ingesting a colostrum replacer or heat-treated (pasteurized) colostrum do not have a high serum GGT concentration, as they do not consume GGT, which is not a component of colostrum replacer and denatured during thermal processing, making this method inadequate for evaluating the colostrum intake in such cases [2].

## 4. Discussion

### 4.1. Improvements in TPI Evaluation

Recent advances and new research for developing new methodologies already provide additional opportunities for TPI assessment in calves, as discussed. Currently, TPI is defined as the absorption of maternal immunoglobulins present in colostrum through the small intestine of the calf during first 24 h after birth [1].

There is evidence that other proteins in colostrum are absorbed by calves, which may expand our understanding of TPI. Zhang et al. [64] showed that colostrum and milk contained several immune system-related proteins, other than immunoglobulins, when studying the proteomes of colostrum and the milk of cows. The results of this study indicate that the most abundant proteins in colostrum and milk have biological functions in the immune system (25%). The functions vary according to the protein, but pass through the presenting antimicrobial activity, as inflammation modulators which participate in the coagulation cascade and the complement system, among other functions. Thus, colostrum transmits not only an adaptive immunity, but also an innate immunity. The specific biological roles of each protein present in the colostrum of calves are not yet fully understood; however, there is strong evidence that they aid the immune systems of calves [64]. Mann et al. [65,82] observed that 261 proteins increased in abundance in the serum of calves evaluated between 0 and 8 h. Among the proteins with increased levels in calf serum, 109 (41%) were present in the colostrum, suggesting that they originated from their absorption. In addition, these proteins were mainly related to the immune response, coagulation, cathelicidins (a class of antimicrobial peptides), the complement system, and acute phase proteins, among others. According to the authors, this reinforced the idea that colostrum played a role in the immunocompetence of the calf that went far beyond immunoglobulin transfer. Thus, the use of innovative techniques for evaluating TPI could help to better understand the complex biological interactions related to passive immunity in calves.

Tóthová et al. [8] observed variations in the globulin fractions between 0 d and 2 d. The total protein contents of (4.11 ± 0.37 and 8.27 ± 0.77 g/dL on 0 d and 2 d, respectively, *p* < 0.001), α1-globulin (1.13 ± 0.08 and 1.26 ± 0.06 g/dL on 0 d and 2 d, respectively, *p* < 0.05), β2-globulin (0.05 ± 0.01 and 0.80 ± 0.18 g/dL on 0 d and 2 d, respectively, *p* < 0.05), and γ-globulin (0.05 ± 0.02 and 2.84 ± 0.55 g/dL on 0 d and 2 d, respectively, *p* < 0.01), but not albumin (2.39 ± 0.30 and 2.08 ± 0.16 g/dL on 0 d and 2 d, respectively, *p* > 0.05), α2-globulin (0.14 ± 0.05 and 0.33 ± 0.05 g/dL on 0 d and 2 d, respectively, *p* > 0.05), and β1-globulin (0.35 ± 0.08 and 0.95 ± 0.25 g/dL on 0 d and 2 d, respectively, *p* > 0.05) were statistically different between 0 d and 2 d, while evaluating the protein profile of seven 0–30 d old calves using electrophoresis. Feitosa et al. [83] also reported statistical differences (*p* < 0.05) in the total protein, β-globulin, and γ-globulin among calves with and without FTPI (4.71 ± 0.84 and 7.02 ± 0.96 g/dL, 0.57 ± 0.19 and 0.93 ± 0.28 g/dL, and 0.65 ± 0.50 and 2.51 ± 0.82 g/dL, respectively), but not (*p* > 0.05) in albumin and α-globulin between the groups (2.52 ± 0.35 and 2.64 ± 3.35 g/dL and 0.97 ± 0.20 and 0.94 ± 0.28 g/dL, respectively). Thus, other proteins present in the colostrum, aside from the immunoglobulins, were also absorbed, which composed the calf serum after absorption and were also associated with the immunity in newborn calves [84]. For example, Tóthová et al. [8] reported that β2-globulin levels increased 10-fold and were associated with lactoferrin, a bioactive component of colostrum with an antimicrobial activity and absorption [1,85,86].

The colostrum of cows also contains several other non-protein components that directly or indirectly affect the immune system of the calf. Amino acids, lactose, fat, maternal cells, vitamins, minerals, and microRNAs are found in colostrum and tend to be in greater quantities than those in milk and may have a biological function in the immune system of the calf [1,87,88,89,90,91]. The consumed glucose from colostrum, for example, increases insulin levels, accelerating the maturation of the somatotropic axis, as a result of stimulating the expression of the hepatic gene for the growth hormone receptors and IGF-1 and the secretion of IGF-1 [90]. In addition to stimulating the growth of the calf, anabolic processes are favored, which may improve the development of the organism as a whole, including the immune system [90]. Miyazaki et al. [91] conducted a pioneering study using the two-dimensional gas chromatography-mass spectrometry (GC × GC)-MS metabolomics method to evaluate the serum metabolite profiles of five male Holstein Friesian calves at eight time points (before colostrum ingestion and at 1, 2, 3, 4, 6, 8, and 12 h after ingestion). GC × GC-MS detected approximately 1400 calf serum metabolites. Eight of these compounds presented temporal changes similar to those changes observed for IgG and corresponded to the oligosaccharides, such as lactose. In addition, some EAA increased in a time-dependent manner in all five of the sampled calves (Thr, Trp, and Met, and a Phe metabolite, phenylacetic acid). The authors suggested that oligosaccharides, including lactose, and EAA, such as Met, Thr, and Trp, may be important colostrum nutrients from neonatal calves, and these compounds may represent potential biomarkers to determine whether calves absorb a sufficient amount of nutrients from colostrum. Therefore, these studies indicate new possibilities for the assessment of TPI, but further studies are needed to better understand the interference of these components in the immunity of the calf.

The direct influence of these colostrum components on the health and survival of calves is conceivable but requires further investigation. Thus, it may be necessary to consider the absorption of other colostrum substances with the potential to assist calf immunity, rather than simply immunoglobulins. Therefore, further studies are warranted to investigate which components directly influence TPI and at what level.

## 5. Conclusions

Due to the importance of proper TPI to calves, the constant monitoring of colostrum management is required on farms. Several methods involving both direct and indirect analyses of serum IgG concentrations are available, with innovative applicability to TPI evaluation. Since, colostrum has several immunological components other than IgG, which are absorbed by the intestines of the calves and most likely present the biological functions in the immune system, new methods could aid in the more complete understanding of TPI in calves. Studies related to the colostrum components are warranted to further investigate the impact of its concentration on TPI and the health and survival of calves.

## Figures and Tables

**Table 1:** The classification of transfer of passive immunity (TPI) assessment methods according to the IgG evaluation method, principle of operation, main characteristics, statistics compared to the gold standard, cost of the method, and possibility of using these methods in the field.

Methods	IgG Evaluation	Main Characteristics of the Method	Statistics Compared to the Gold Standard	Using These Methods in the Field
RID	Direct	Gold standard, classic method, but time-consuming	-	No
Turbidimetric Immunoassays	Direct	Fast, does not require reagents from the user, can be expensive	R^2^ = 0.98 [3]	Yes
ELISA	Direct	Time-consuming, requires bench equipment and often needs repetition	R^2^ = 0.97 [44]; r = 0.90 [9]	No
Electrophoresis	Direct	Commonly used for serum protein fractionation, highly widespread in laboratories, and requires bench equipment	89% accuracy [6]	No
CE	Direct	Fast, precise, accurate, fully automated, compatible with small sample volumes, inexpensive, and requires bench equipment	r = 0.97 [9]	No
Proteomics	Direct	Used to separate and individually quantify sample proteins; expensive and not widespread in laboratories as it requires bench and expensive equipment	We found no such studies	No
AP-MALDI-MS	Direct	Ease to operate, fast, precise, robust, compatible with small sample volumes, inexpensive, and requires bench equipment	We found no such studies	No
Spectroscopic method	Direct	Simple and fast measurement that requires little or no sample preparation; expensive and requires expensive equipment	r = 0.93 [11]	Not yet
STIGA	Direct	High potential to be a direct analysis of IgG in calves for use in the field	R^2^ = 0.83–0.94 [13]	Not yet
Biochemical analysis of total proteins and fractions	Indirect	Highly widespread in laboratories and cheap; requires bench equipment	r = 0.83 [45]	Not yet
TP by refractometry	Indirect	Cheap, simple, fast, and can be used on farms, uses portable equipment	r = 0.93 [19]; r = 0.41 [17]	Yes
BRIX by refractometry	Indirect	Used in the field to assess both TPI and colostrum quality, uses portable equipment	r = 0.93 [19]	Yes
Zinc sulfate turbidity test	Indirect	Cheap, simple, fast, and can be used on farms	R^2^ = 0.78 [44]	Yes
Turbidity test for sodium sulfite	Indirect	Cheap, simple, fast, and can be used on farms	88% accuracy [46]	Yes
Biochemical serum GGT analysis	–	Evaluates only unpasteurized maternal colostrum intake, requires bench equipment	r = 0.57 [14]	Not yet

RID, radial immunodifusion; IgG, immunoglobulin G; ELISA, enzyme-linked immunosorbent assay; CE, Capillary electrophoresis; AP-MALDI-MS, Atmospheric pressure matrix-assisted laser desorption/ionization by mass spectrometry; STIGA, split trehalase IgG assay; GGT, gamma glutamyltransferase; TPI, transfer of passive immunity.
